# How accurate are witnesses of first suspected seizures in recalling semiology at clinically relevant timepoints? A UK experimental study with a pilot intervention

**DOI:** 10.1111/epi.18624

**Published:** 2025-09-06

**Authors:** Adam J. Noble, Steven Lane, Paul New, Harriet Cope, Chloe Foley, Holly Lynn Williams, Laszlo Sztriha, Graham Powell, Markus Reuber, Anthony G. Marson

**Affiliations:** ^1^ Department of Public Health, Policy and Systems University of Liverpool Liverpool UK; ^2^ Department of Health Data Science University of Liverpool Liverpool UK; ^3^ Department of Psychological Sciences University of Liverpool Liverpool UK; ^4^ Department of Neurology King's College Hospital National Health Service Foundation Trust London UK; ^5^ Neurology Department Walton Centre National Health Service Foundation Trust Liverpool UK; ^6^ Department of Neuroscience, School of Medicine and Population Health University of Sheffield Sheffield UK; ^7^ Department of Pharmacology and Therapeutics University of Liverpool UK

**Keywords:** accurate diagnosis, first seizures, recall, waiting times, witnesses

## Abstract

**Objective:**

A key diagnostic challenge at “first seizure” clinic appointments is determining whether the reported event was epileptic. Witness accounts are often critical, yet such appointments typically occur weeks after the event. Guidelines recommend review within 2 weeks. Wait times are however often longer, with a median of 7 weeks in countries such as the UK. The accuracy of witness recall at these clinically relevant intervals and whether their confidence predicts accuracy have never been determined. This study addressed these fundamental questions. It also piloted a potential intervention: whether asking witnesses a set of systematic questions immediately after viewing a suspected seizure improves recall at follow‐up, compared to the usual free recall approach used by first responders.

**Methods:**

In this UK‐based experimental study, adults (≥18 years old) viewed a video of an epileptic seizure and were randomized into four conditions: A (immediate free recall + 2‐week follow‐up), B (immediate free recall + 7‐week follow‐up), C (immediate systematic questions + 2‐week follow‐up), and D (immediate systematic questions + 7‐week follow‐up). The primary outcome was accuracy on 15 standardized questions addressing key semiological features, scored against consensus ratings from five neurologists.

**Results:**

Of a representative sample of 304 participants, 295 (97%) fully viewed the video, and 94.7% completed follow‐up. At 2 weeks, participants answered 54.4% of questions correctly—only 3.9% (95% confidence interval [CI] = .52–7.3) more than those at 7 weeks. Confidence was poorly correlated with accuracy. Immediate systematic questioning improved later recall by 6.7% (95% CI = 3.3–10.0). A definitive trial of this intervention would require 926 participants.

**Significance:**

This is the first evidence on the accuracy of witness recall at clinically relevant intervals. Recall is modest even within recommended timeframes and declines only slightly by 7 weeks. Witness confidence does not predict accuracy. Immediate structured questioning may enhance later recall and thus support seizure diagnoses.

**Key points**

- Witness recall was experimentally tested 2 and 7 weeks after seeing a seizure, mirroring typical “first seizure” clinic wait times.
- Witnesses could answer 63% of semiology questions correctly immediately after viewing a seizure video; this dropped to 54% at 2 weeks.
- Recall accuracy declined only slightly further by 7 weeks, with witnesses answering ~50% of questions correctly.
- Witness confidence in their recall did not reliably indicate how accurate their recall was.
- Systematic questioning of witnesses immediately after seeing the seizure improved later recall by ≤10%. It may be a useful intervention.

## 1 INTRODUCTION

Seizures and seizure‐like events are common with 1 in 25 people experiencing at least one epileptic seizure by age 85 years.^1^ Guidelines emphasize the importance of timely referral to a specialist following a first suspected seizure.^2^, ^3^, ^4^, ^5^ However, in many countries patients can face significant waits.^6^, ^7^, ^8^, ^9^, ^10^, ^11^ UK guidelines recommend specialist evaluation within 2 weeks,^4^, ^5^ but current wait times range from 4 to 12 weeks, with a median of 7 weeks.^6^

Although the causes of delays may vary across health systems, the consequences appear consistent, namely, potentially increased morbidity and mortality.^12^, ^13^, ^14^ Less is known, however, about how wait times impact the accuracy of suspected seizure witness testimony. A systematic literature search found no relevant evidence (Appendix S1).

This evidence gap is concerning, because clinicians often draw on witness accounts to help determine whether a first event was epileptic. Certain features that theoretically can be observed and recalled by witnesses—such as side‐to‐side head movements, ictal eye closure, sudden shaking or stiffening, and signs of impaired self‐control—contribute significantly within statistical models in distinguishing epilepsy from other conditions.^15^, ^16^, ^17^, ^18^ Witness reports may also offer insights into seizure severity and localization.

Studies have assessed witness accuracy immediately after viewing a video of seizure and raised the possibility that their recall is imperfect.^19^, ^20^, ^21^, ^22^ Although important, such findings do not clarify how accurate witness recall is at the later follow‐up times seen or recommended in practice. Understanding recall accuracy over such periods is essential not only for clinicians, but also for policymakers considering the diagnostic consequences of increasing wait times.

Another unresolved question is how witness confidence relates to recall accuracy. This is also important for clinicians to know because, in the absence of an objective measure, confidence may be used as a proxy for reliability and guide the weight assigned to it. In forensic settings, jurors find witness testimony delivered with confidence to be more persuasive, regardless of accuracy.^23^

These uncertainties highlight the need for an experimental study to assess how seizure witness recall changes over time. Such a study could involve participants viewing a seizure video and being randomized to recall the event after either 2 or 7 weeks, while also rating their confidence in their recollections. This framework also presents an opportunity to explore potential interventions to enhance memory retention.

One potentially promising, low‐cost intervention is to change how witnesses are managed in the prehospital setting, immediately after a suspected seizure. Most referred to a specialist after a first suspected seizure initially present to the emergency services.^6^, ^7^, ^8^, ^9^ Currently, first responders such as paramedics may ask the witness informal questions, but they are not systematically interviewed until much later. A more effective approach might involve systematic questioning by first responders, as evidence from forensic psychology indicates that responding to questions—verbally or in writing—within 24 h can significantly reduce memory decay and vulnerability to misinformation.^24^ For this reason, some policing authorities already recommend such techniques in certain contexts (e.g., College of Policing^25^).

Before such an intervention can be tested in a definitive trial with “real‐world” seizure witnesses in the prehospital setting, key design parameters must first be resolved. This includes identifying and resolving any key concerns with the acceptability of study procedures, assessing how recall accuracy varies across individuals and over time, and estimating whether immediate systematic questioning improves later recall compared to initial free recall. The latter is critical for calculating any such trial's sample size. In such cases, guidelines recommend conducting a pilot focused on feasibility and informing the design of a definitive trial.^26^

Accordingly, the current experimental study aimed to:

1. Evaluate the accuracy of seizure witness recall at 2 versus 7 weeks after viewing a seizure event;
2. Assess the relationship between witness confidence and actual recall accuracy; and
3. Gather participant feedback on study acceptability and estimate whether systematic questioning postevent improves follow‐up accuracy compared to free recall and describe its implications for the sample size of a definitive trial.

## 2 MATERIALS AND METHODS

### 2.1 Design

The experiment was conducted via the online platform Qualtrics, where participants remotely viewed items and responded to questionnaires at different assessment points. A 2 × 2 factorial between‐subjects design was employed. The independent variables were (1) time between stimulus video and final recall (2 weeks vs. 7 weeks) and (2) immediate management to secure initial recall (“free recall” vs. “systematic questions”). Participants were randomly assigned to one of four conditions (A, B, C, D) in a 1:1:1:1 ratio. The dependent variable was accuracy of witness recall at follow‐up.

### 2.2 Ethics

The University of Liverpool's Institute of Population Health Research Ethics Committee approved the study (Ref: 14688). All participants provided informed consent.

### 2.3 Recruitment

Participants were recruited via Prolific in January 2025. They were informed the study involved eyewitness accounts but unaware of its aims or hypotheses. Prolific distributed invitations to a stratified sample based on age, sex, and ethnicity, reflecting UK Census data. Participants received GBP 4 for the baseline questionnaire and GBP 3 for the follow‐up. Further recruitment details are in Appendix S2.

### 2.4 Eligibility criteria

Participants were UK residents aged ≥18 years, able to complete a survey in English, with normal or corrected vision and hearing, and internet access with audio. Exclusion criteria included terminal illness, severe psychiatric conditions, or inability to provide informed consent.

### 2.5 Measures

#### 2.5.1 Witness recall

Recall of the seizure was assessed at baseline (conditions C + D) and follow‐up (all conditions) using 15 items from Erba et al.'s suspected seizure witness questionnaire (Table 1).^15^, ^18^ Items focused on seizure semiology. Five UK consultant neurologists with epilepsy expertise^27^ reviewed the video and completed the same items to provide a consensus‐based judgment, against which participant answers were compared (Appendix S3).

**TABLE 1:** Items used to assess participant recall and the accuracy of the recall of key groups.

Overall recall	Comparisons of interest			
	Participants grouped by time of follow‐up		Participants grouped by immediate management	
	2 weeks [conditions A + C], *n* = 147	7 weeks [conditions B + D], *n* = 141	Free recall [conditions A + B], *n* = 142	Systematic recall [conditions C + D], *n* = 146
% correct				
Mean (SD)	54.4 (12.8)	50.5 (16.4)	49.1 (14.4)	55.8 (14.5)
Median (IQR)	53.3 (46.7–60.0)	53.3 (40.0–60.0)	53.3 (40.0–60.0)	53.3 (46.7–66.7)
Minimum–maximum	13.3–86.7	13.3–80.0	13.3–80.0	13.3–86.7
Raw				
Mean (SD)	8.2 (1.9)	7.6 (2.5)	7.4 (2.2)	8.4 (2.2)
Median (IQR)	8.0 (7.0–9.0)	8.0 (6.0–9.0)	8.0 (6.0–9.0)	8.0 (7.0–10.0)
Min‐max	2.0–13.0	2.0–12.0	2.0–12.0	2.0–13.0

Individual recall items	Correct, *n* (%)	Rank	Correct, *n* (%)	Rank	Correct, *n* (%)	Rank	Correct, *n* (%)	Rank
1. Did you observe any of the following at the very beginning of the seizure?…	51 (34.7)	12	53 (37.6)	12	41 (28.9)	12	63 (43.2)	12
2. Did the patient shake or stiffen?	140 (95.2)	2	115 (81.6)	2	124 (87.3)	2	131 (89.7)	2
3. How did the shaking or stiffening start?	101 (68.7)	=4	65 (46.1)	=9	68 (47.9)	8	98 (67.1)	6
4. How did the patient shake or stiffen?	32 (21.8)	13	21 (14.9)	14	16 (11.3)	14	37 (25.3)	13
5. Did the shaking or stiffening stop abruptly and then start back up during the seizure?	89 (60.5)	8	94 (66.7)	6	88 (62.0)	5	95 (65.1)	7
6. How did the shaking or stiffening stop?	117 (79.6)	3	95 (67.4)	5	95 (66.9)	4	117 (80.1)	3
7. Did the patient's head turn strongly to one side?	100 (68.0)	6	80 (56.7)	7	77 (54.2)	7	103 (70.5)	5
8. Was there movement of the head from side to side?	61 (41.5)	11	60 (42.6)	11	54 (38.0)	11	67 (45.9)	11
9. During the seizure were the patient's eyes closed or open?	72 (49.0)	9	65 (46.1)	=9	66 (46.5)	9	71 (48.6)	10
10. Did you see any of the following during the seizure?…	69 (46.9)	10	69 (48.9)	8	59 (41.5)	10	79 (54.1)	9
11. Do you see any of the following during the seizure?…	26 (17.7)	14	23 (16.3)	13	26 (18.3)	13	23 (15.8)	14
12. Did the patient fall during the seizure?	142 (96.6)	1	120 (85.1)	1	126 (88.7)	1	136 (93.2)	1
13. How long did the actual seizure activity last?	101 (68.7)	=4	99 (70.2)	4	87 (61.3)	6	113 (77.4)	4
14. Right at the end of the seizure activity, did you see any of the following?…	95 (64.6)	7	100 (70.9)	3	112 (78.9)	3	83 (56.8)	8
15. Right at the end of the seizure activity, what was their level of consciousness and awareness?	3 (2.0)	15	8 (5.7)	15	6 (4.2)	15	5 (3.4)	15

*Note:* The response options for all questions ‐ including 1, 10, 11 and 14 ‐ are provided in full in Table S3.3 in Appendix S3. Questions for each recall item were ranked for each condition/assessment point according to the proportion of correct responses elicited (with rank 1 = highest proportion of correct responses; an "=" in the ranking column indicates a joint/tied position).

Abbreviation: IQR, interquartile range.

#### 2.5.2 Confidence

At follow‐up, participants rated their overall confidence in their recall on a 1–9 Likert scale (1 = “pure guess” to 9 = “absolutely certain”) in response to the question, “How confident are you in your answers about the seizure in the video?”

#### 2.5.3 Acceptability

At follow‐up, participants answered three adapted^28^ questions about study experience:

1. Would you agree to participate again if time suddenly went backward?
2. Please explain why or why not.
3. Any improvements to the study?

### 2.6 Procedure

At baseline, participants ensured their device sound was on and provided demographic information. They were then shown a 97‐s video^29^ of an adult male having an uncomplicated focal‐to‐bilateral tonic–clonic seizure lasting ~90 s. This type was chosen because it commonly prompts referral to “first‐seizure” clinics.^30^, ^31^

Afterward, participants completed a 9‐min filler task designed to simulate ambulance response time in European nations (e.g., Noble et al.^32^) and thus the typical time between witnessing a suspected seizure and first being asked to recall it in any detail. The task set included an attention check, question about the device used, and then simple distractor questions.^33^ Following this, participants were randomized into one of four conditions. Conditions A and B asked for free recall descriptions; conditions C and D involved answering the 15 systematic questions from Erba et al.'s questionnaire.^18^ Participants were then asked about personal/familial seizure experience.

Participants were told they would complete a follow‐up after 2 weeks (conditions A + C) or 7 weeks (conditions B + D). A single reminder was sent to persons not initially responding to the invitation to complete their follow‐up questionnaire.

### 2.7 Analysis

With no prior evidence on changes in seizure witness recall accuracy over time, the sample size was based on estimating key parameters for a future trial. A target of 50 per condition was deemed sufficient to estimate variation and effect, increased by 33% to allow for missing data and possible dropouts, yielding 65 per condition. Previous recommendations suggest 24–50 participants are adequate for pilot studies.

Analyses were conducted using Stata (v18.0). Descriptive statistics summarized baseline characteristics and compared (without formal statistical tests) participants' age, sex, and ethnicity with UK Census data.

Recall accuracy—defined as the proportion of correctly answered questions—was calculated for conditions C and D at baseline, and for all conditions at follow‐up. The effect of time was estimated by comparing follow‐up recall of the 2‐week conditions (A + C) with the 7‐week conditions (B + D). The effect of immediate management was assessed by comparing free recall conditions (A + B) with systematic recall conditions (C + D). Results are reported as mean differences with 95% confidence intervals (CIs).

Recall questions were ranked by the proportion of correct responses across conditions. Accuracy is also separately reported for three potentially key diagnostic items from the full questionnaire: Q3 (onset of shaking/stiffening), Q8 (side‐to‐side head movements), and Q9 (ictal eye closure).^15^, ^16^, ^18^

Spearman rank correlations assessed the relationship between recall accuracy and confidence.

With a significance level (alpha) of 5% and variability determined from the current study in follow‐up recall across all conditions, we used G*Power (3.1.9.7) to calculate the sample size needed for a definitive trial to have 90% power to detect a difference determined by the lower limit of the 95% CI for the estimated mean difference between conditions A + B and C + D.

## 3 RESULTS

### 3.1 Participant recruitment, allocation, watching of stimulus video, and completion time

Of 322 invited individuals, 320 consented to participate. Of these, 307 were randomized (Appendix S4): 77 to condition A, 76 to condition B, 77 to condition C, and 77 to condition D. After randomization, one participant did not complete the baseline questionnaire, and two submissions from repeat participants were excluded, leaving 304 with complete baseline data (condition A = 77, B = 75, C = 76, D = 76).

Nearly all participants (*n* = 295, 97.0%) watched the video in full, with *n* = 298 (98.0%) passing an attention check (Table 2). There were no obvious differences between conditions in the proportions of participants fully watching the video, device used, or distractor task engagement.

**TABLE 2:** Characteristics of all participants recruited, by randomized condition and in comparison to UK adult population.

Characteristic	Consented but withdrew or removed, *n* = 16^a^	Consented & completed baseline, *n* = 304	UK representative, *n* = 305^b^	Condition			
				A, *n* = 77	B, *n* = 75	C, *n* = 76	D, *n* = 76
Age, years							
Median (IQR)	54.5 (32.5–64.5)	48 (33–60)	‐	47 (29–60)	47 (32–60)	50.5 (36.8–62)	48 (32–59.8)
Range	23–70	19–80	‐	20–74	20–80	19–80	19–78
18–24	1 (7.1)	33 (10.9)	34 (11.1)	10 (13.0)	10 (13.3)	3 (3.9)	10 (13.2)
25–34	3 (21.4)	50 (16.4)	51 (16.7)	13 (16.9)	11 (14.7)	13 (17.1)	13 (17.1)
35–44	2 (14.3)	48 (15.8)	49 (16.1)	10 (13.0)	15 (20.0)	12 (15.8)	11 (14.5)
45–54	1 (7.1)	53 (17.4)	51 (16.7)	18 (23.4)	7 (9.3)	15 (19.7)	13 (17.1)
55–100	7 (50.0)	120 (39.5)	120 (39.3)	26 (33.8)	32 (42.7)	33 (43.4)	29 (38.2)
Missing	2	0	0	0	0	0	0
Sex, *n* (%)							
Female	4 (36.4)	158 (52.0)	158 (51.8)	39 (50.6)	38 (50.7)	40 (52.6)	41 (53.9)
Male	7 (63.6)	144 (47.4)	147 (48.2)	37 (48.1)	36 (48.0)	36 (47.4)	35 (46.1)
Prefer not to say	0	2 (.7)	0	1 (1.3)	1 (1.3)	0	0
Missing	0	0	0	0	0	0	0
Ethnicity, *n* (%)							
White	2 (40.0)	250 (82.5)	250 (82.0)	64 (83.1)	70 (93.3)	59 (77.6)	57 (76.0)
Asian	0	23 (7.6)	25 (8.2)	5 (6.5)	2 (2.7)	6 (7.9)	10 (13.3)
Black	3 (18.8)	10 (3.3)	10 (3.3)	4 (5.2)	1 (1.3)	4 (5.3)	1 (1.3)
Mixed	0	10 (3.3)	10 (3.3)	2 (2.6)	1 (1.3)	4 (5.3)	3 (4.0)
Other	0	10 (3.3)	10 (3.3)	2 (2.6)	1 (1.3)	3 (3.9)	4 (5.3)
Missing	11	1	‐	0	0	0	1
Seizure experience, *n* (%)							
Experienced seizure &/or dx. seizure disorder	0 (100.0)	19 (6.3)	‐	6 (7.8)	5 (6.8)	3 (4.0)	5 (6.8)
Family/friend seizure &/or dx. seizure disorder, *n* (%)	0 (100.0)	84 (28.0)	‐	29 (37.7)	22 (29.7)	16 (21.3)	17 (23.0)
Seen someone have a suspected seizure in person, *n* (%)	2 (66.7)	130 (43.3)	‐	40 (51.9)	30 (40.5)	28 (37.3)	32 (43.2)
Missing	13	4	‐	0	1	1	2
Watched video completely, *n* (%)							
Yes	‐	295 (97.0)	‐	76 (98.7)	72 (96.0)	73 (96.1)	74 (97.4)
Missing	‐	0	‐	0	0	0	0
Device watched on, *n* (%)							
Phone	‐	81 (26.6)	‐	19 (24.7)	24 (32.0)	14 (18.4)	24 (31.6)
Tablet	‐	24 (7.9)	‐	6 (7.8)	2 (2.7)	9 (11.8)	7 (9.2)
Computer	‐	199 (65.5)	‐	52 (67.5)	49 (65.3)	53 (69.7)	45 (59.2)
Missing	‐	0	‐	0	0	0	0
Attention check, *n* (%)							
Passed	‐	298 (98.0)	‐	75 (97.4)	71 (94.7)	76 (100.0)	76 (100.0)
Missing	‐	0	‐	0	0	0	0
Distractor questions answered [of 137]							
Median (IQR)	‐	45 (39.0–69.0)	‐	45 (39–64.5)	46 (25–67)	44 (39–69.8)	48 (40–70)
Range	‐	7–137	‐	8–137	7–134	19–137	16–112
Missing	‐	0	‐	0	0	0	0
Free recall engagement^c^							
Median number of words used (IQR)	‐	48.5 (31–72.5)	‐	52 (32.5–79.5)	48 (30–67)	‐	‐
Range	‐	5–219	‐	16–201	5–219	‐	‐
Missing	‐	0	‐	0	0	‐	‐

*Note:* Condition A, immediate free recall, 2‐week follow‐up; condition B, immediate free recall, 7‐week follow‐up; condition C, immediate systematic recall, 2‐week follow‐up; condition D, immediate systematic recall, follow‐up 7 weeks.

Abbreviations: dx., diagnosed; IQR, interquartile range.

^a^ Comprised of 13 participants who consented but withdrew prior to randomization, one person who withdrew immediately following randomization, and two submissions deemed to have arisen from someone who had already taken part in the study.

^b^ Age, sex, and ethnicity estimates are derived from the 2021/2022 UK Census data.

^c^ Task only requested of the participants in conditions A and B.

The median time it took participants to complete the baseline questionnaire was similar for those for whom it involved free recall (16.1 min, interquartile range [IQR] = 14.0–19.5) and for those for whom it involved responding to the systematic questions (16.7, IQR = 14.7–20.1). Participants in conditions A + B typed a median of 48.5 words (IQR = 31.0–72.5) in response to the free recall question.

### 3.2 Participant demographics

Median participant age was 48.0 years (IQR = 33–60, range = 19–80); 152 (52.0%) were female (Table 2). Participants were comparable in age, sex, and ethnicity to the UK adult population, and participants in the different conditions were similar in demographics and previous seizure experiences. Of the 300 of 304 participants who answered questions about previous seizure experiences, 130 (43.3%) reported having witnessed a suspected seizure in person.

### 3.3 Participant retention

Two hundred eighty‐eight (94.7%) participants returned their follow‐up questionnaire with completed responses to the 15 items testing their recall. It took them a median of 4.5 min (IQR = 3.3–6.8) to complete. Retention was minimally higher in conditions with a 2‐week follow‐up (A + C; 93.5%–98.7%) compared to those with a 7‐week follow‐up (B + D; 93.3%–93.4%; Appendix S4).

### 3.4 Accuracy of witness descriptions, variation, and confidence

#### 3.4.1 Effect of scheduled follow‐up time

##### 3.4.1.1 Two‐week recall compared to 7‐week recall

**FIGURE 1:**
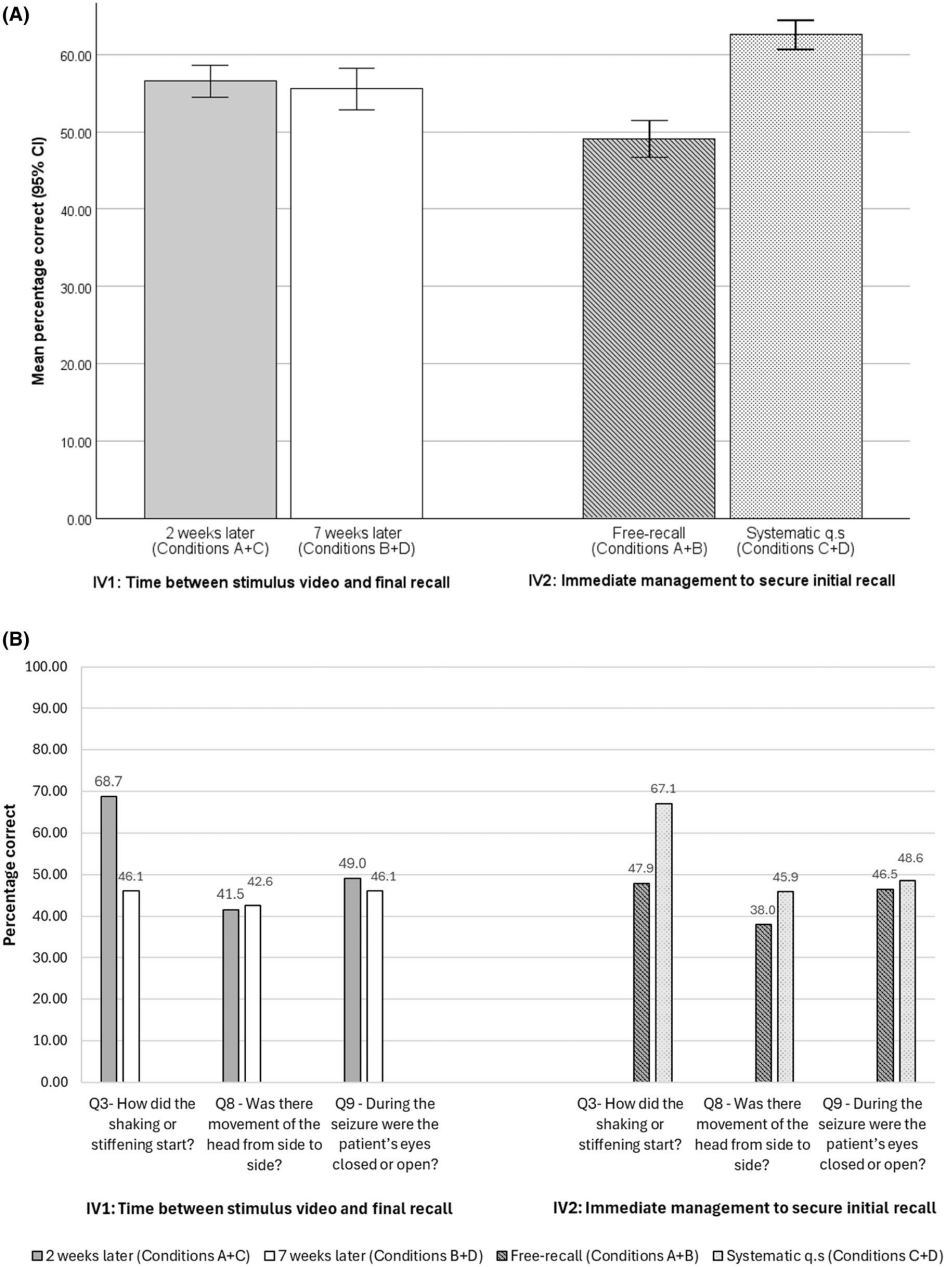
(A) Mean proportion of correctly answered questions at follow‐up, by follow‐up timing (2 vs. 7 weeks) and whether the person received immediate systematic questioning. (B) Percentage of participants by follow‐up point, and by immediate systematic questioning status, who correctly answered questions about specific seizure features: onset of shaking/stiffening, head movement, and eye closure. CI, confidence interval; Condition A, immediate free recall, 2‐week follow‐up; Condition B, immediate free recall, 7‐week follow‐up; Condition C, immediate systematic recall, 2‐week follow‐up; Condition D, immediate systematic recall, follow‐up 7 weeks; IV, independent variable; q.s, questioning.

Participants followed‐up at 2 weeks (conditions A + C) answered a significantly higher proportion of questions correctly (mean = 54.4, SD = 12.8) than participants followed‐up at 7 weeks (conditions B + D; mean = 50.5, SD = 16.4; mean difference = 3.9, 95% CI = .52–7.34; Figure 1A). In absolute terms, this difference was small. It equated to participants answering a mean of .60 (95% CI = .08–1.10) more questions correctly (Table 1; Appendix S5 presents accuracy for each condition).

The items that participants were least and most accurate on assessed at 2 weeks and at 7 weeks were the same (Q15 and Q12, respectively; Table 1). Participants followed‐up at 2 weeks gave more correct responses than those followed up at 7 weeks on the potentially key items regarding manner of onset of any shaking/stiffening (Q3, 68.7% vs. 46.1%) and ictal eye closure (Q9, 49.0% vs. 46.1%), but not on the item regarding head movement (Q8, 41.5 vs. 42.6%; Figure 1B).

Participants followed‐up at 2 weeks expressed slightly higher confidence in their recall (mean = 5.6, SD = 1.4) than those followed‐up at 7 weeks (mean = 4.8, SD = 1.8). Among participants followed up at 2 weeks, confidence held only a small positive association with recall accuracy (ρ[147] = .19). Confidence was moderately associated with accuracy for participants followed up at 7 weeks (ρ[141] = .44).

##### 3.4.1.2 Immediate recall compared to recall at 2‐ or 7‐week follow‐up

Participants who were asked systematic questions (conditions C + D) immediately after watching the video answered a mean of 62.5% (SD = 11.8) of them correctly. Participants in both conditions answered significantly more questions correctly immediately after watching the video than at their 2‐ or 7‐week follow‐up (Appendix S5). The largest difference was seen in condition D (7‐week follow‐up), with a mean difference in the proportion answered correctly of 10.1 (95% CI = 6.36–13.73). For participants in condition C (2‐week follow‐up), the mean difference was 4.4 (95% CI = 1.39–7.32).

#### 3.4.2 Effect of immediate management to secure initial recall

Participants who were initially asked systematic questions (conditions C + D) answered more questions correctly at follow‐up than participants who were not (Figure 1A). Specifically, they answered a mean of 55.8% (SD = 14.5) of questions correctly compared to 49.1% (SD = 14.4) by those asked to complete free recall (conditions A + B; mean difference = 6.7, 95% CI = 3.3–10.0). This equated to them answering a mean of 1.0 (95% CI = .50–1.51) more questions correctly. Both groups were least and most accurate on the same items (Table 1).

Condition C + D participants outperformed condition A + B participants on the questions related to the onset of shaking/stiffening (Q3, 67.1% vs. 47.9%), side‐to‐side head movements (Q8, 45.9% vs. 38.0%), and eye closure (Q9, 48.6% vs. 46.5%; Figure 1B).

### 3.5 Participant feedback

All 288 participants who provided follow‐up data completed the feedback questionnaire. Of these, *n* = 285 (99.0%) said they would “definitely” (*n* = 239, 83%) or “probably” (*n* = 46, 16%) take part again. In justifying their answer, no participant commented negatively about the recall questions.

### 3.6 Sample size calculation for definitive trial

With a pooled SD of 14.74 and using the lower bound of the mean difference between conditions A + B and C + D of 3.3, 421 participants for each condition would be required, giving a total sample size of 842. To ensure sufficient participants at the end of the trial, we suggest conservatively inflating the sample size by ~10% to 926 to allow for potential attrition.

## 4 DISCUSSION

### 4.1 Recall accuracy

Our study is the first to assess suspected seizure witness recall accuracy at clinically relevant timepoints and also clarifies the relevance, for recall, of increasing wait times seen in some countries for appointments. We found 2 weeks after viewing a seizure—when “first seizure” clinic appointments are often recommended to occur—witnesses correctly answered only 54% of questions on semiology. This fell to ~50% at 7 weeks. These findings highlight that even within current referral timeframes, recall is limited, and delays may further degrade accuracy.

The recall accuracy we observed at follow‐up shows that previously available estimates—based on studies assessing recall immediately after observing a seizure^19^, ^20^, ^21^, ^22^, ^34^—would not have been reliable guides for clinicians in “first‐seizure” clinics, because they would have overestimated likely accuracy. In our study, participants in conditions C and D correctly answered approximately 63% of questions immediately after viewing the video, closely matching the 65% reported by Muayqil et al.^19^ Their study, the only other to recruit from the general population, involved Saudi Arabian adults watching a similar seizure video to ours and being asked 17 semiology questions immediately afterward.

We also found only a small correlation between confidence and accuracy at 2 weeks, and a moderate one at 7 weeks—novel findings that align with broader forensic psychology literature.^35^ Clinicians should thus not infer accuracy from witness confidence. Emerging research suggests alternative markers—such as the presence of “effort cues” (e.g., hedges, pauses) within a person's answers—might be more helpful. This merits examination within the context of seizure witness recall.^36^

Our study found witnesses recall gross motor features (e.g., shaking, falling) more accurately than finer or more abstract features (e.g., shaking onset, awareness). Notably, features often considered diagnostically valuable—such as ictal eye closure or side‐to‐side head movements—were poorly recalled, with typically <50% accuracy at both timepoints. Limitations in witness recall could thus plausibly impact diagnostic accuracy if over‐relied on. Our finding that first‐time witnesses may not reliably recall diagnostically important details aligns with previous studies in which experienced seizure witnesses—family and friends—described the typical seizures of someone they knew, with their accounts compared to video recordings of those seizures.^37^, ^38^

Considering our results alongside prior work, a tentative forgetting curve emerges; recall is higher immediately after observing the event, it declines at 2 weeks, and loss begins to plateau by 7 weeks. This broadly mirrors Ebbinghaus's classic forgetting curve,^39^ and suggests related evidence from cognitive psychology—particularly on the value of repetition and spacing to optimize memory—could be drawn on to help further understand seizure witness recall.

Our experimental design was robust and its parameters driven to make its estimates of accuracy clinically applicable. Some factors, however, could have acted to produce estimates differing slightly from real‐world accuracy. First, participants viewed a video of a stranger and were forewarned (a condition for ethical approval). Although the latter is likely to only have minimally affected recall,^40^ the emotional intensity of viewing the seizure was likely lower for our participants than for witnesses seeing a seizure in a known person.^41^ It is unclear how this might have impacted recall. Stress does affect memory, but the relationship is complex. Gering et al.^42^ suggest an inverted‐U relationship; moderate stress enhances memory, whereas too little or too much impairs it. To avoid unintentionally improving recall, we thus did not seek to use further experimental techniques to induce additional stress.

Second, we used a fixed‐response questionnaire as the outcome measure. This differs from the open interview format typically used in “first seizure” clinics. Clinical interviews may generate different responses to questions on semiology. However, our approach enabled consistent quantification of accuracy across a large sample and aligns with most prior studies.^19^, ^21^, ^22^, ^34^ Moreover, we caution against assuming interviews automatically generate more accurate recall. Forensic psychology research shows interviewer questions can—even unintentionally—distort witness memory.^43^

### 4.2 Enhancing recall and the case for a definitive trial

Given modest witness accuracy at clinically relevant follow‐up intervals, identifying interventions to enhance recall is a clinical priority. We piloted one intervention: systematic questions delivered immediately postevent, within the typical window of interaction with first responders. Evaluated against the APEASE^44^ framework (Acceptability, Practicability, Effectiveness, Affordability, Side‐effects, Equity)—which can be used to prioritize candidate interventions—systematic questioning shows promise. Of particular note is that our study provides an estimate suggesting potential effectiveness, with follow‐up recall being up to 10% higher among those who received systematic questioning versus those who did not. Power calculations indicate a definitive trial would require 926 participants—a feasible target in most countries, given suspected seizures account for ~2.5% of ambulance calls (in European countries).^45^ Moreover, our piloting indicated good acceptability, with no participants reporting issues with the questions.

We contend that there might be a case for ambulance services embedding the 15‐item seizure witness questionnaire (or a similar tool) into assessment protocols for suspected seizures, regardless of the outcome of any future trial on its impact on later witness recall. Currently, seizure‐specific minimum data items are not embedded within the electronic report forms used by ambulance clinicians (at least not in the UK); what is recorded is left to clinician discretion and often captured in free text. As a result, potentially valuable prehospital records available to specialists in “first‐seizure” clinics are inconsistent and may lack key information, including witness accounts. Embedding such a tool into electronic report forms could enhance diagnostic accuracy by improving the quality of information available to specialists, support their conversations with witnesses, and help mitigate the risk of key witnesses being unavailable at the time of clinic assessment. In our study, witnesses completed the measure in approximately 4 min—well within the ~30‐min average on‐scene time of paramedics.^32^

### 4.3 Strengths and limitations

Strengths include adherence to reporting guidelines^46^ and programmatic randomization that produced demographically balanced conditions. Accuracy was rigorously assessed and based on the judgments of five neurologists—more than in most previous studies.^19^, ^20^, ^21^, ^22^ Unlike earlier work,^20^, ^21^, ^22^ given it could generate unrepresentative estimates of recall accuracy, we avoided intentionally recruiting participants because they had medical training.^34^, ^47^

Potential limitations include the use of Prolific for recruitment, which may reduce external validity as participants self‐selected. However, studies suggest it yields high‐quality, reproducible data.^48^ Recall and intervention effects were assessed using a single seizure type; future work should thus explore other types. Finally, to minimize burden, we asked participants to provide an overall rating of confidence in their recall, rather than for each individual question.

## 5 CONCLUSIONS

Witness recall of seizure events is only moderately accurate at intervals commonly recommended and seen in clinical practice. Confidence does not reliably indicate accuracy. Systematic questioning immediately after the event may enhance later recall and warrants definitive evaluation.

## AUTHOR CONTRIBUTIONS

**Adam J. Noble:** Conceptualization; methodology; formal analysis; investigation; resources; data curation; writing—original draft; project administration and supervision. **Steven Lane:** Methodology; formal analysis; data curation; writing—review & editing. **Paul New:** Investigation; methodology; writing—review & editing. **Harriet Cope:** Investigation; methodology; writing—review & editing. **Chloe Foley:** Investigation; methodology; writing—review & editing. **Holly Lynn Williams:** Investigation; methodology; writing—review & editing. **Laszlo Sztriha**: Methodology; resources; writing—review & editing. **Graham Powell:** Methodology; resources; writing—review & editing. **Markus Reuber:** Methodology; resources; writing—review & editing. **Anthony G. Marson:** Conceptualization; methodology; resources; writing—review & editing.

## CONFLICT OF INTEREST STATEMENT

None of the authors has any conflict of interest to disclose. We confirm that we have read the Journal's position on issues involved in ethical publication and affirm that this report is consistent with those guidelines.

## Supporting information


Appendix S1.

Appendix S2.

Appendix S3.

Appendix S4.

Appendix S5.

## Data Availability

Data are available upon reasonable request. All requests for data sharing should be submitted to the corresponding author for consideration. Access to anonymized data may be granted following review.
