# Supplementary material for: How accurate are witnesses of first suspected seizures in recalling semiology at clinically relevant timepoints? A UK experimental study with a pilot intervention
Source: Epilepsia. 2025 Sep 6;66(12):4795–808. doi: 10.1111/epi.18624 (PMC12779316; doi:10.1111/epi.18624)
Supplement: Supplementary file 1 — Appendix S1. [file EPI-66-4795-s003.docx]

**Appendix S1** Systematic literature search: Methods and summary of studies

**Methods**

*Identification*

Searches were made of Scopus, Medline and Wed of Science from inception until 29/09/2024. The following search terms (article titles and abstracts) were used and modified for the different databases:

1. witness OR bystander OR informant OR caller
2. AND recall OR memory OR testimony OR remember OR account
3. AND seiz* OR epilep* OR convuls* OR "non epileptic attack" OR "NEAD" OR "functional neurological disorder" OR "FND" OR "loss of consciousness"

*Eligibility and screening*

To be eligible an article had to be reported in English, be published in a peer-reviewed journal and the study needed to have examined the accuracy of first-time suspected seizure witness recall.

Titles/abstracts for identified articles were screened for eligibility by two reviewers (AN, HLW), removing duplicates and obviously irrelevant studies. Full texts versions of the articles that they both agreed as ostensibly eligible were accessed and further reviewed for eligibility by both reviewers. Agreement between them was high (98%) and all discrepancies were resolved through discussion.

**Results**

The selection process and reasons for exclusion are shown in the PRISMA flow chart Figure S1 below. Ultimately 3 eligible studies were identified. They are summarised in the Table S1.

They all examined witness recall immediately after watching a video and each used study specific items to assess recall. Two studies involved watching videos of epileptic seizures, whilst the other involved watching a video of an epileptic seizure or reflex syncope. One study was conducted in England, one in the Netherlands and one in Saudia Arabia. None of the studies assessed longer-term recall accuracy.


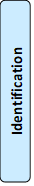


Total identified (n = 268)

Web of Science
(n = 130)

Medline
(n = 35)

Scopus

(n=103)


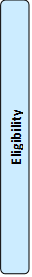


Duplicate records (n =115)

Unique records

(n = 153)

Records excluded based on title and abstract
(n =146)

Full text articles examined

(n = 7)

Records excluded after full article review

(n =4)

*Reasons:*

Not-first time witnesses= 2

Recall accuracy not tested= 2


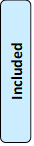


Eligible studies identified

(n = 3)

**FIGURE S1** PRISMA Flow chart showing identification and selection assessment

**TABLE S1**  Summary of findings from eligible studies identified by systematic search

| **Author** | **Location** | **Whose recall was assessed?** | **Event/s observed** | **Sample size** | **Was video played without warning?** | **How long after viewing event was accuracy assessed?** | **How was recall assessed?** | **How was accuracy determined?** | **Attention check included?** | **Main findings** |
| --- | --- | --- | --- | --- | --- | --- | --- | --- | --- | --- |
| Mannan & Wieshmann^1^ | England | Students and junior doctors (medical students, junior doctors on neurological ward, non-medical students) | Individually shown video of a focal seizure that generalize | 20 | No | Immediately | Participants provided written accounts of event.  . | Accounts were evaluated based on eight semiological features. Participants received one point for each correct recall and lost one point for each incorrect observation. Two neurologists independently scored the accounts, with high inter-rater agreement. | No | ●Mean total score was 2.7 (range −2 to 6). ●Unresponsiveness and lateralising  Features often missed.  ●Erroneously described  features included ‘patient rolled over‘, ‘agitated’ or ‘arms flopped about’.  ● Left and right were sometimes confused.  ●Junior doctors  and medical students who had received epilepsy teaching  did not score consistently higher  than non-medical students and medical students without  epilepsy teaching. |
| Thijs et al.^2^ | Netherlands | Students (university; psychology) | Lecture theatre cohorts were shown a video of either a generalized tonic–clonic seizure or reflex syncope. | 229 | Yes | Immediately | The study included 11 self-administered, study-specific questions on semiology. | Consensus view of 4 neurologists who could view video multiple times. | Yes | ●Epileptic seizure: Of all responses to items observable in the video, 60% were correct, 18% were erroneous, and  22% were “I do not know” responses. ●Reflex syncope: Of all responses to items observable  in the video, 44% were correct, 28% were erroneous,  and 29% were “I do not know” responses.  ●For neither video was the total number of correct observations affected by sex, previous experience with similar attacks, or question order. |
| Muayqil et al.^3^ | Saudia Arabia | Adults aged ≥17 from general population | Individually shown video on laptop computer of a  focal seizure that generalized OR partial seizure that did not. | 172 participants randomized at the time of recruitment to view 1 video. | No | Immediately | Study-specific, interviewer-administered questions included 17 on semiology for generalizing seizures and 13 for non-generalizing seizures. | Consensus view of 3 neurologists. | No | ●Generalising seizure: Participants on average answered 64.7% of questions correctly. Highest items recalled correctly were ‘involvement of head’ (89.9%) and involvement of an arm (84.8%). Most incorrect responses were for whether movement of head was vertical (18.5%) and presence of vocalizations (29.7%).  ●Non-generalising seizure: Mean percentage correctly answered was 54.7%; Highest items recalled correctly were ‘involvement of head’ (93.8%) and whether there was a focal start to the seizure (88.8%). Most incorrect responses were for which leg/s moved (25%) and type of leg movement (28.8%). |

**REFERENCES**

1. Mannan JB, Wieshmann UC. How accurate are witness descriptions of epileptic seizures? Seizure. 2003 Oct;12:444-447.

2. Thijs RD, Wagenaar WA, Middelkoop HA, Wieling W, van Dijk JG. Transient loss of consciousness through the eyes of a witness Neurology. 2008 Nov 18;71:1713-1718.

3. Muayqil TA, Alanazy MH, Almalak HM, Alsalman HK, Abdulfattah FW, Aldraihem AI, et al. Accuracy of seizure semiology obtained from first-time seizure witnesses BMC Neurol. 2018 Sep 1;18:135.
