# Supplementary material for: How accurate are witnesses of first suspected seizures in recalling semiology at clinically relevant timepoints? A UK experimental study with a pilot intervention
Source: Epilepsia. 2025 Sep 6;66(12):4795–808. doi: 10.1111/epi.18624 (PMC12779316; doi:10.1111/epi.18624)
Supplement: Supplementary file 2 — Appendix S2. [file EPI-66-4795-s004.docx]

**Appendix S2** Additional details on recruitment process

***Further details on recruitment process:***

Participants were recruited via the platform Prolific (<https://www.prolific.com/>). People interested in taking part in research register on this platform. Upon registering, they answer pre-screening questions to allow their eligibility (e.g., age) for different studies to be assessed. Prolific then notifies persons, via its internal systems, of adverts for studies that they appear eligible for and might be interested in taking part in. Platforms such as Prolific have been used by prior seizure and epilepsy related studies wanting access to large general population samples (e.g.,^1-4^).

For the present pilot study, Prolific sent a notification of the study and its advert to persons whose screening responses indicated they resided in the UK and had a current age of 18 or above. The title used for the study was “A Study of your Perspective as A Witness: The SPAW study”.

As per the conditions of ethical approval, the notification sent to ostensibly eligible persons and the accompanying advert included: 1) included a content warning highlighting to them that the study covered a potentially sensitive topic; 2) explicitly stated to those who expressed an interest that it involved watching a video of a seizure; and 3) highlighted to readers that for some people watching a video of such a medical event may be upsetting and encouraged them to consider this before taking part.

Those who remained interested in the study were be directed to a study survey page, hosted by Qualtrics where they could access a Participant Information Sheet, the contact details for the study team, and if appropriate, complete a consent form and proceed to enter the study and complete the baseline survey.

***Further details on Prolific:***

Most persons taking part in projects via Prolific originate from countries with developed economies in the Organisation for Economic Co-operation and Development (OECD) group. It has demonstrated an ability to routinely support studies requiring a range of sample sizes. In 2022, 9,345 studies were conducted that recruited samples of ≥ 500 participants.^5^ Evidence is also available to show that Prolific and other such platforms can provide access to a broad population and that results from studies recruiting via it are comparable to those obtained by studies using more classical routes. ^6, 7^ An additional advantage worth noting is that the way a study is run on Prolific reduces the likelihood of multiple submissions by participants since they can only receive a maximum of one payment for a single study. Prolific has clear guidelines for the handling of submissions and defines a minimum fixed payment per unit of time required to complete an experiment that is also communicated to participants when they sign up to the platform. Participants have a quick and risk-free option for withdrawing their consent at any time during a study without it affecting any ‘acceptance/reputation’ score.

**REFERENCES**

1. Silva AB, Leonard MK, Oganian Y, D'Esopo E, Krish D, Kopald B, et al. Interictal epileptiform discharges contribute to word-finding difficulty in epilepsy through multiple cognitive mechanisms Epilepsia. 2023 Dec;64:3266-3278.

2. Simonsson O, Goldberg SB, Chambers R, Osika W, Long DM, Hendricks PS. Prevalence and associations of classic psychedelic-related seizures in a population-based sample Drug Alcohol Depend. 2022 Oct 1;239:109586.

3. Tran EB, Vonk JMJ, Casaletto K, Zhang D, Christin R, Marathe S, et al. Development and validation of a nonverbal consensus-based semantic memory paradigm in patients with epilepsy J Int Neuropsychol Soc. 2024 Aug;30:671-679.

4. Holmes E, Bourke S, Plumpton C. Attitudes towards epilepsy in the UK population: Results from a 2018 national survey Seizure. 2019 Feb;65:12-19.

5. Tomczak J, Gordon A, Adams J, Pickering JS, Hodges N, Evershed JK. What over 1,000,000 participants tell us about online research protocols Front Hum Neurosci. 2023;17:1228365.

6. Douglas BD, Ewell PJ, Brauer M. Data quality in online human-subjects research: Comparisons between MTurk, Prolific, CloudResearch, Qualtrics, and SONA PLoS One. 2023;18:e0279720.

7. Peer E, Brandimarte L, Samat S, Acquisti A. Beyond the Turk: Alternative platforms for crowdsourcing behavioral research Journal of Experimental Social Psychology. 2017;70:153-163.
