# Supplementary material for: How accurate are witnesses of first suspected seizures in recalling semiology at clinically relevant timepoints? A UK experimental study with a pilot intervention
Source: Epilepsia. 2025 Sep 6;66(12):4795–808. doi: 10.1111/epi.18624 (PMC12779316; doi:10.1111/epi.18624)
Supplement: Supplementary file 4 — Appendix S4. [file EPI-66-4795-s001.docx]

**Appendix S4** Diagram of flow of participants through the study


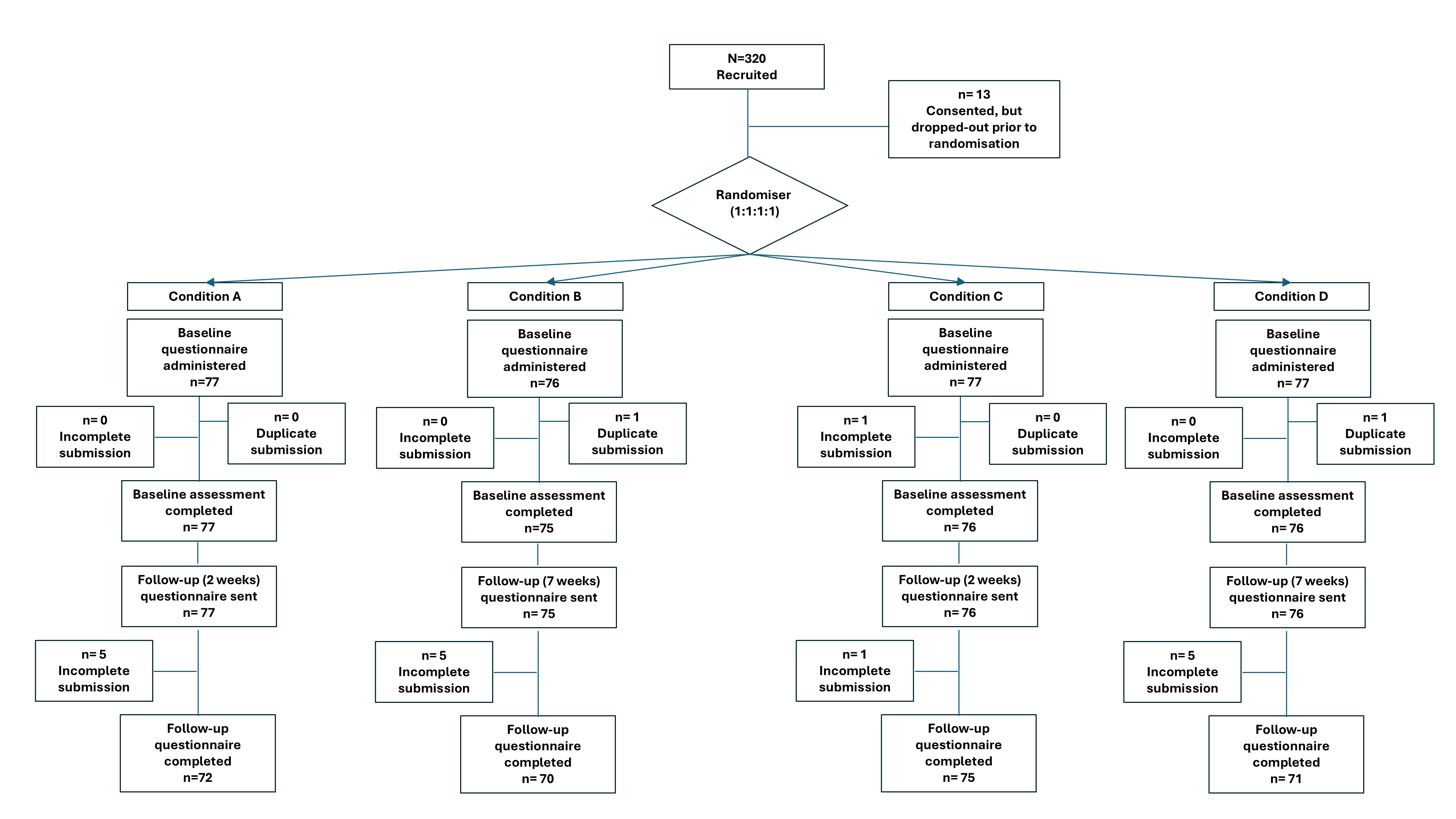


***Notes:*** Condition A, Immediate free-recall, 2-week follow-up; B, Immediate free-recall, 7-week follow-up; C, Immediate systematic recall, 2-week follow-up; D, Immediate systematic recall, follow-up 7 weeks.
