# Supplementary material for: How accurate are witnesses of first suspected seizures in recalling semiology at clinically relevant timepoints? A UK experimental study with a pilot intervention
Source: Epilepsia. 2025 Sep 6;66(12):4795–808. doi: 10.1111/epi.18624 (PMC12779316; doi:10.1111/epi.18624)
Supplement: Supplementary file 5 — Appendix S5. [file EPI-66-4795-s005.docx]

**Appendix S5** The accuracy of recall participants in the individual conditions at different assessment points and their confidence

|  | **CONDITION** | | | | | | | | | | | | | | | |
| --- | --- | --- | --- | --- | --- | --- | --- | --- | --- | --- | --- | --- | --- | --- | --- | --- |
|  | **A** | | | | **B** | | | | **C** | | | | **D** | | | |
|  | ***Baseline***  *(Free recall)*  (N=77) | | ***Follow-up***  *(2 weeks)*  (N=72) | | ***Baseline***  *(Free recall)*  (N=75) | | **Follow-up**  *(7 weeks)*  (N=70) | | **Baseline**  *(Systematic recall)*  (N=76) | | **Follow-up**  *(2 weeks)*  (N=75) | | **Baseline**  *(Systematic recall)*  N=76) | | **Follow-up**  *(7 weeks)*  (N=71) | |
| **Overall recall** | | | | | | | | | | | | | | | | |
| % Correct  Mean (SD)  Median (IQR)  Min-Max | n/a  n/a  n/a | | 50.8 (11.7)  53.3 (41.7-60.0)  13.3-80.0 | | n/a  n/a  n/a | | 47.2 (16.6)  53.3 (40.0-60.0)  40.0-80.0 | | 61.9 (11.7)  60.0 (53.3-71.7)  33.3-86.7 | | 57.8 (13.0)  60.0 (53.3-66.7)  20.0-86.7 | | 63.2 (11.9)  66.7 (53.3-73.3)  53.3-73.3 | | 53.6 (15.7)  53.3 (46.6-66.7)  13.3-80.0 | |
| Raw  Mean (SD)  Median (IQR)  Min-Max | n/a  n/a  n/a | | 7.6 (1.8)  8.0 (6.3-9.0)  2.0-12.0 | | n/a  n/a  n/a | | 7.1 (2.5)  8.0 (6.0-9.0)  2.0-12.0 | | 9.3 (1.8)  9.0 (8.0-10.8)  5.0-13.0 | | 8.7 (1.9)  9.0 (8.0-10.0)  3.0-13.0 | | 9.5 (1.8)  10.0 (8.0-11.0)  5.0-13.0 | | 8.0 (2.4)  8.0 (7.0-10.0)  2.0-12.0 | |
| **Individual recall items** | | | | | | | | | | | | | | | | |
|  | ***Correct n (%)*** | ***Rank*** | ***Correct***  ***n (%)*** | ***Rank^a^*** | ***Correct n (%)*** | ***Rank*** | ***Correct***  ***n (%)*** | ***Rank^a^*** | ***Correct***  ***n (%)*** | ***Rank^a^*** | ***Correct***  ***n (%)*** | ***Rank^a^*** | ***Correct***  ***n (%)*** | ***Rank^a^*** | ***Correct***  ***n (%)*** | ***Rank^a^*** |
| 1. Did you observe any of the following at the very beginning of the seizure? | n/a | n/a | 19 (26.4) | 12 | n/a | n/a | 22 (31.4) | 12 | 30 (39.5) | 12 | 32 (42.7) | 11 | 35 (46.1) | 12 | 31 (43.7) | =11 |
| 2. Did the patient shake or stiffen? | n/a | n/a | 68 (94.4) | 2 | n/a | n/a | 56 (80.0) | 2 | 75 (98.7) | =1 | 72 (96.0) | 2 | 73 (96.1) | 1 | 59 (83.1) | 2 |
| 3. How did the shaking or stiffening start? | n/a | n/a | 42 (58.3) | =6 | n/a | n/a | 26 (37.1) | 10 | 51 (67.1) | 9 | 59 (78.7) | 5 | 47 (61.8) | 9 | 39 (54.9) | 9 |
| 4. How did the patient shake or stiffen? | n/a | n/a | 10 (13.9) | 14 | n/a | n/a | 6 (8.6) | 14 | 18 (23.7) | 13 | 22 (29.3) | 13 | 23 (30.3) | 13 | 15 (21.1) | 13 |
| 5. Did the shaking or stiffening stop abruptly and then start back up during the seizure? | n/a | n/a | 43 (59.7) | 5 | n/a | n/a | 45 (64.3) | =5 | 56 (73.7) | 6 | 46 (61.3) | 7 | 58 (76.3) | 8 | 49 (69.0) | 5 |
| 6. How did the shaking or stiffening stop? | n/a | n/a | 50 (69.4) | 4 | n/a | n/a | 45 (64.3) | =5 | 70 (92.1) | 3 | 67 (89.3) | 3 | 59 (77.6) | =5 | 50 (70.4) | 4 |
| 7. Did the patient’s head turn strongly to one side? | n/a | n/a | 42 (58.3) | =6 | n/a | n/a | 35 (50.0) | 7 | 55 (72.4) | 7 | 58 (77.3) | 6 | 54 (71.1) | 7 | 45 (63.4) | 7 |
| 8. Was there movement of the head from side to side? | n/a | n/a | 29 (40.3) | 11 | n/a | n/a | 25 (35.7) | 11 | 36 (47.4) | 11 | 32 (42.7) | =11 | 43 (56.6) | 10 | 35 (49.3) | 10 |
| 9. During the seizure were the patient’s eyes closed or open? | n/a | n/a | 32 (44.4) | 9 | n/a | n/a | 34 (48.6) | 8 | 44 (57.9) | 10 | 40 (53.3) | 8 | 36 (47.4) | 11 | 31 (43.7) | =11 |
| 10. Did you see any of the following during the seizure? | n/a | n/a | 30 (41.7) | 10 | n/a | n/a | 29 (41.4) | 9 | 65 (85.5) | 5 | 39 (52.0) | 9 | 68 (89.5) | 4 | 40 (56.3) | 8 |
| 11. Do you see any of the following during the seizure? | n/a | n/a | 14 (19.4) | 13 | n/a | n/a | 12 (17.1) | 13 | 4 (5.3) | =14 | 12 (16.0) | 14 | 18 (23.7) | 14 | 11 (15.5) | 14 |
| 12. Did the patient fall during the seizure? | n/a | n/a | 69 (95.8) | 1 | n/a | n/a | 57 (81.4) | 1 | 75 (98.7) | =1 | 73 (97.3) | 1 | 72 (94.7) | 2 | 63 (88.7) | 1 |
| 13. How long did the actual seizure activity last? | n/a | n/a | 41 (56.9) | 8 | n/a | n/a | 46 (65.7) | 4 | 69 (90.8) | 4 | 60 (80.0) | 4 | 70 (92.1) | 3 | 53 (74.6) | 3 |
| 14. Right at the end of the seizure activity, did you see any of the following? | n/a | n/a | 58 (80.6) | 3 | n/a | n/a | 54 (77.1) | 3 | 54 (71.1) | 8 | 37 (49.3) | 10 | 59 (77.6) | =5 | 46 (64.8) | 6 |
| 15. Right at the end of the seizure activity, what was their level of consciousness and awareness? | n/a | n/a | 2 (2.8) | 15 | n/a | n/a | 4 (5.7) | 15 | 4 (5.3) | =14 | 1 (1.3) | 15 | 5 (6.6) | 15 | 4 (5.6) | 15 |
| **CONFIDENCE IN RECALL** | | | | | | | | | | | | | | | | |
| Mean (SD) | n/a | | 5.54 (1.33) | | n/a | | 4.71 (1.76) | | n/a | | 5.71 (1.40) | | n/a | | 4.97 (1.75) | |

***Notes:*** n, number; n/a, not applicable as participants in this condition were not asked to complete this measure. IQR, interquartile range; Min, minimum; Max, maximum; SD, standard deviation.

^a^ Questions for each recall item were ranked for each condition/assessment point according to the proportion of correct responses elicited (with rank 1= highest proportion of correct responses).
